# Role of Emotional Appraisal in Episodic Memory in a Sample of Argentinean Preschoolers

**DOI:** 10.3389/fpsyg.2019.02556

**Published:** 2019-12-05

**Authors:** Eliana Ruetti, María Soledad Segretin, Verónica Adriana Ramírez, Sebastian J. Lipina

**Affiliations:** ^1^Unidad de Neurobiología Aplicada, Centro de Educación Médica e Investigación Clínica Norberto Quirno (CEMIC), Buenos Aires, Argentina; ^2^Facultad de Psicología, Universidad de Buenos Aires, Buenos Aires, Argentina

**Keywords:** emotions, episodic memory, appraisal, individual differences, preschoolers

## Abstract

Emotional processing and episodic memory are closely related throughout childhood development. With respect to emotional episodic memory, available evidence shows that the consolidation of information is accompanied by an arousal that generates longer duration and persistence of the memory representations. In the case of early stages of development (i.e., first 5 years), it is less clear how these associations emerge and are modulated by individual and environmental factors. In this study, 116 4- to 5-years old Argentinean children from different socio-environmental contexts (i.e., favorable or unfavorable living conditions at home), performed a task of visual emotional memory in which they observed a set of 15 images with variable emotional valences (negative, neutral, and positive). The child’s task was to appraise each image using one of the following three possible valences: (a) drawings of faces with smiles (positive valence), (b) drawings of faces with tears and round mouth with edges down (negative valence), or (c) drawings of faces with horizontal mouth (neutral valence). Five years-old children exhibited greater accuracy appraisal. Individual differences in emotional accuracy appraisal allowed us to observe different performances in free recall of negative visual images. Accuracy appraisal did not vary between children with respect to gender, living conditions at home, or language ability. Seven to ten days after the emotional appraisal children were asked to tell the experimenter all the images they remembered (variables of interest: free recall of negative, positive, or neutral images). Results showed individual (age) differences. Specifically, 5-years-old children evoked more images than 4-years-old children. These findings contribute to the understanding of emotional memory in early developmental stages and raise the need to include emotional appraisal in the assessment of episodic memory.

## Introduction

People’s lives are influenced by their experiences. Memory encodes, stores, and allows information to be evoked for future use (Baddeley, 1999). The memory of past events begins to develop early in life and changes throughout the preschool and kindergarten years (Bauer et al., 2012). An example of this is the number of events or items that children can retrieve, the length of that span, and the association of memory representations and processes on external or internal keys for their retrieval (Bauer et al., 2010; Ghetti and Bunge, 2012).

There are several studies in adults, which emphasize the interaction between memory and emotions (Justel et al., 2013). In this sense, emotional events are better remembered than the most trivial ones (Christianson, 1992; Quas and Lench, 2007; Brainerd et al., 2010). For example, a childhood accident is remembered accurately, but dinner a week ago is unlikely to be remembered. However, the direction in which this differential effect occurs is not always clear. Emotions would function as a filter system of selection of events that are going to be stored in memory in a more lasting way (Rodríguez et al., 2004). Specifically, emotional memory is the result of storing information that was accompanied by stressful and/or activating factors through which that memory could have been fixed more easily. It is a type of long-term memory about information with emotional valence, positive or negative (Bermúdez-Rattoni and Prado-Alcalá, 2001; Justel et al., 2013; Guzmán-Ramos et al., 2018; Siller-Pérez et al., 2018).

Some studies analyzed the factors involved in the consolidation and retrieval of emotional experiences in children (Cordon et al., 2013). Specifically, age seems to be an influential factor in these memories because there are marked differences in the amount of information that children remember and the accuracy with which they do it (Goodman et al., 1994; Merritt et al., 1994; Salmon et al., 2002; Brainerd et al., 2010; Leventon et al., 2014; Langnes et al., 2018). Because of this, age is a reliable predictor of memory performance, both for emotional (Goodman et al., 1997) and non-emotional information (Courage and Cowan, 2008). For example, children who are 4- and 5-years-old can encode and evoke more specific and integrated emotional information than children who are 3-years-old (Goodman et al., 1997; Brainerd et al., 2008; Wang, 2008).

As children develop their linguistic abilities, an increase in their performance in memory tasks occurs, which indicates the importance of verbal language when analyzing changes in memory development. Studies in children of 3- to 5-years old, in which they provided information on events of daily life, found that older children remembered more information than younger ones and used conditional verb tenses in their stories due to the greater development of their language ability (Hudson and Nelson, 1986; Fivush et al., 2003, 2008). In addition to age, the emotions that accompanied the performance during memory tasks can be another modulator. Emotionally significant experiences that occurred during childhood can be retained and remembered for a significant length of time (Cordon et al., 2013). In the same sense, Channell and Barth (2013) found that the accuracy of memories about emotional information was significantly related to the emotional knowledge of the children, suggesting that this association could not be explained simply by maturation, but individual differences also played an important role in memory processing.

When considering possible individual differences in the emotional appraisal, gender could be considered an intervening factor. There are few studies in children considering this variable. However, in adults there is some consensus in the literature about a greater reactivity of women to negative valence stimuli (Lang et al., 1993; Bradley and Lang, 2000). Specifically, a greater reactivity of the amygdala was verified before threatening images in women than in men (Canli et al., 2002). This reaction was also founded in emotional expression recognition in women with anxiety disorders (Ohrmann et al., 2010). In addition, on the assessment of affective images, there is a higher tendency in adult women to value negative images as such than men (Bradley and Lang, 2000). In accordance to these results, various physiological factors (such as skin conductance and shock boosted by fear) suggest that girls aged 7 to 10-years are more sensitive to initiate a physiological defensive mobilization than boys (McManis et al., 2011), because they also tend to value more negative images as such. Regarding development of the emotional recognition of facial expressions in children from 4 to 9-years old, an effect of gender was identified, with more recognition in the girls’ performance (Widen and Russell, 2010). In another study on recognition of expressions of varied intensity in children from 4 to 18-years old the same study also founded that girls recognize facial expressions better than men, especially those of anger and disgust (Montirosso et al., 2010).

In relation to gender, ratings of emotional valence and arousal did not differ significantly as a function of participant gender (Cordon et al., 2013). Because of that gender differences did not examined in the children’s emotional memory (Leventon and Bauer, 2016). Furthermore, due to gender differences in the socialization of emotion (e.g., Brody and Hall, 2010) and mixed evidence for gender differences in appraisal measured in children (e.g., Dennis and Hajcak, 2009), are necessary to examine gender differences in emotional appraisal and emotional memory expression.

On the other hand, socio-environmental conditions also can modulate the performance of children in learning and memory tasks (Principe et al., 2017). For example, Tessler and Nelson (1994) found that the conversation between mothers and children during a visit to a museum influenced the content of the memories in children of 3-years-old; none of them reported aspects of the visit that had not been discussed. In other study, Haden et al. (2001) organized three events in the homes of 30-, 36-, and 42-months-old children and recorded mothers and children’s conversations during shared activities. The children recalled more of the activities that had been discussed with their mothers compared with those that were only spoken of by the mother or that were not discussed at all. In another study, children’s memories of external situations with emotional valence (e.g., natural disasters, abuse episodes), which were usually negative, enhanced their performance during a memory task (Haden et al., 2001).

As previously mentioned, negative or positive stimuli are generally processed differently from non-emotional (neutral) stimuli, and they are remembered more clearly over time in adults and children (Bradley et al., 1992; Cahill and Alkire, 2003; Cordon et al., 2013). However, it is not yet clear in what sense the emotional valence of events is associated with memory processing in preschoolers. Emotional appraisal can be defined as the process through which different responses are generated from the same event, based on subjective assessment of events or stimuli (Nieto and Delgado, 2006; Kuppens et al., 2012; Moors, 2013, 2017; Moors and Scherer, 2013; Fernando et al., 2015; Parsafar and Davis, 2018; Scherer and Fontaine, 2018). People constantly perform emotional assessments of events and stimuli according to what it implies for their own well-being, objectives, and concerns (Kuppens et al., 2012). These appraisal responses are considered the closest psychological determinants of emotions (Ellsworth and Scherer, 2003; Kuppens et al., 2012), so these would be one of the results of the assessment (Ellsworth and Scherer, 2003; Parsafar and Davis, 2018).

There is agreement among several researchers that emotions include different components, such as appraisal, the tendency to act or to cope, behavioral actions, physiological changes, and subjective expressions (Scherer and Fontaine, 2018). In this sense, Russell (2009) proposed a two-dimensional theory of emotion that is composed of appraisal and intensity (valence and arousal) of a stimulus, where various phenomena occurred in each emotional episode (i.e., changes in facial expressions, vocal tone, functioning of the peripheral nervous system, emotional appraisal, behavior, subjective experience, and emotional regulation; Scherer and Fontaine, 2018). The appraisal process would be integrated into emotional knowledge, which also would include aspects such as understanding of emotions, expression of emotions, and the subsequent recovery of that information to be integrated into memories (Channell and Barth, 2013; Fidalgo et al., 2018).

However, children and adults may react differently to the same stimulus (Davies and Logie, 1993; Roseman and Smith, 2001; Nieto and Delgado, 2006; Fernando et al., 2015). This implies, on the one hand, that appraisal is crucial to defining emotional processing and its consequent response rather than the properties of the stimulus itself (Fernando et al., 2015). On the other hand, this also means that this appraisal would be modulated by individual factors (e.g., age, gender, temperament, development of language skills) and socio-environmental factors (e.g., demographic and economic characteristics of the family context) (Davies and Logie, 1993; Fivush and Baker-Ward, 2005; Nieto and Delgado, 2006; Wang, 2008). Despite the increase in the number of studies that address these issues, there are few that focus on the emotional appraisal of visual stimuli in preschoolers and its association with memory processes (Kuppens et al., 2013).

The aim of this paper is to analyze emotions and memory associations through an emotional memory paradigm in preschoolers. In this study, 4 and 5 years old children from different sociodemographic contexts (favorable or unfavorable) were tested in one task to analyze their processing to visual images with emotional information (negative and positive valences) or non-emotional images (neutral valence). Specifically, we analyzed the role of the emotional appraisal accuracy on the later expression of emotional memory. Additionally, we analyzed whether gender, socio-environmental conditions, and the children’s language skills modulated their performances in accuracy appraisal. *Accuracy of appraisal* is the variable that can be considered, which arises as a result of the comparison between the children appraisal and the task appraisal as defined by researchers (valence of each stimulus). According to the literature, it is expected: (a) that age and the memory will be associated, that is, that 5-years-old children will evoke more images than those of 4-years-old (Goodman et al., 1997; Cordon et al., 2013); (b) differences in the episodic memory will meet: greater number of images will be recalled for emotional (negative and positive) compared to neutral content (Bradley et al., 1992; Cordon et al., 2013); and (c) modulation of the emotional free recall by the emotional appraisal and the socio-environmental conditions at home (Ellsworth and Scherer, 2003; Kuppens et al., 2012; Principe et al., 2017). With regard to gender (differences between girls and boys) and language skills (low, moderate and high performance) associations with emotional memory performances (based on children’s own appraisal, and on task appraisal) will be explored.

## Materials and Methods

### Participants

A sample of 116 children (56 girls and 60 boys) that were 4 (*n* = 50) or 5 (*n* = 66) years old was conformed. The children were students at one of three kindergartens from the Autonomous City of Buenos Aires: two private schools (*n* = 54) and one public school (*n* = 62). The children included in the sample had no developmental disorders in terms of their perinatal and postnatal health history.

### Ethical Considerations

Informed consents were obtained from parents/caregivers, and ethical approval was obtained from the Ethical Review Committee of CEMIC (Directorate of Research. Av. Galván 4102, 1st Floor, C1431FWO, Autonomous City of Buenos Aires. Protocol N° 961). The study was conducted according to American Psychological Association ethical standards and international and national children rights laws. Once the authorization was obtained from the school institutions where the activities were carried out, information meetings were organized for parents in which they had the opportunity to interact with the members of the research group, who informed them about the objectives and activities of the study, and then gave them a written information consent with the same content. Consequently, they were invited to participate and to authorize the participation of their children, for which the signing of informed consent for each of the evaluations was a necessary condition.

### Tasks and Assessment Procedures

Two tasks with were administered in this study. The first was emotional memory task with two component.

Component A. *Emotional appraisal*. This component assesses the attribution of emotional expressions to emotional images. The instrument consisted of two sets of stimuli. The first set was composed of 15 images with different valences (five negative, five positive, and five neutral) that was obtained from Development Affective Photographs System to children (DAPS; Cordon et al., 2013). All the images were in color, they had people (i.e., children, women), animals (i.e., dogs, cockroach), objects (i.e., book, cup), or people performing actions (i.e., children playing with a ball, girl jumping the rope). These images were presented in counterbalanced order on a tablet or on a notebook. The second set consisted of three images (in white and black) with emotional expressions (happy, sad, or neutral faces). Children observed the images of the first set and then had to choose one expression to show how they felt when they saw the images. The three images of the faces appeared on a sheet, which the children had available while they watched each image. Each photograph lasted on the screen until the children chose a face, and decided to move forward with the images, that is, the time varied among the children, but lasted approximately 2 s. The faces with emotional expressions were available all the time for the children to decide which one to choose. The participants did not have a time limit to decide. The choice was made by pointing with one finger, one of the three faces with emotional expressions. Before beginning the task, the participants observed two example images. The variable of interest was children appraisal response to images that had different valences (negative, positive, or neutral). This variable can be defined as the attribution of valence by each child to each visual stimuli with different emotional contents.

Component B. *Emotional memory*. This component consisted of two phases. First, children were asked to evoke immediately (immediate emotional memory) the appraised images of set 1 described above (this variable was obtained to control that children codified the visual information, but it was not incorporated as a variable of interest in the study). Then, after a delay of 7–10 days, children were again request to evoke those images (emotional memory test). The memory test consisted in the evocation of visual information through free recall. Children said words or phrases about images that they observed during the task. The variables of interest were the free recall of negative, positive, or neutral images, which was defined as the number of images of each type evoked after a time interval since the occurrence of the task presentation.

The second task was Navarra’s oral language test (PLON; Aguinaga et al., 1991) was used to control the relationship between memory capacity and linguistic expression. The sub-scales of understanding, expression, and social knowledge were administered. For 4 years old children, in the understanding sub-scale, a sheet of 10 images was presented and the child was asked to point to six objects, one at a time. For the expression sub-scale, a sheet of seven images was presented and the child was asked to name each one while pointing them out. For the social knowledge sub-scale, children were asked to answer four questions about basic needs (e.g., “What do you do when you are sleepy? What do you do when you are thirsty? What do you do when you got cold?”). For 5 years old children, in the understanding sub-scale, images were presented to the child, and he/she was asked to point to objects by their use (e.g., “Point out something that is good for playing”). In the expressive sub-scale, images were presented and the child was asked what the object was used for. I In the social knowledge sub-scale, three pictures of children performing actions were presented, and children were asked what they were doing. Variables of interest were the scores obtained in each sub-scale, which were categorized according to level into 0 (low: scores equal to zero in the subscales); 1 (moderate: scores 1–5 in the subscales); and 2 (high: scores >6 in the subscales).

Tasks were administered in two different sessions of 20–30 min each one. In the first session, the emotional appraisal component was administered. In the second session, the emotional memory component and the PLON task were administered.

### Characterization of Living Conditions

A Socio-Economic Level questionnaire (NES) was used to obtain child health history, socio-economic, and socio-environmental information on their living conditions at home. This information was obtained from the report of the families in individual interviews with the researchers. This scale was used in previous studies in Argentina (Lipina et al., 2004, 2005; Segretin et al., 2009, 2014; Prats et al., 2017). NES was also used to evaluate parents’ education and occupational levels, housing and overcrowding, conditions, and to identify indicators of unsatisfied basic needs (UBN; Boltvinik, 1995). Scores were assigned directly to mothers and fathers for educational and occupational backgrounds; however, only the higher score was considered for the total scores. For housing conditions, scores were assigned based on type of dwelling, floor, water, bathroom, ceiling, external walls, and home property. Living conditions at home were considered *unfavorable* when at least one of the indicators of UBN was present; if none of these indicators was present, living conditions at home were considered *favorable*. Thus two groups were formed, and the unfavorable condition showed lower scores of the indicators (Table 1).

**TABLE 1:** Mean and standard deviation of education, occupation, housing, and overcrowding scores of living conditions homes.

	**Favorable (*n* = 32)**		**Unfavorable (*n* = 40)**			
	***M***	***SD***	***M***	***SD***	***Z***	***P***
Education	11	5.36	7	3.12	−6.08	0.000
Occupation	9	2.73	5	2.51	−5.96	0.000
Housing	12	0.00	10	1.90	−4.80	0.000
Overcrowding conditions	9	2.67	8	2.45	−3.26	0.001

*Scores obtained from NES. The sample size is lower than the total sample (*N* = 116), however, no significant differences were found in the cognitive performance (emotional appraisal and emotional memory) of the children who have socio-environmental information, and children who do not (*p* ≥ 0.05).*

### Data Analysis

First, we performed univariate analyses of the variables of interest, which included the mean, median, standard deviation, standard error, and sample size for each age group. Then, normality and homoscedasticity of the variables were evaluated. When the parametric requirement were not met, trigonometric transformations were applied to the variables, and assumptions were re-tested with the transformed variables. Non-parametric statistics were used, because some assumptions were indicators of UBN not met. The Mann–Whitney *U*-test was used for independent samples that considered the age (4 or 5 years), gender (girls, boys), and living conditions (favorable, unfavorable), and emotional appraisal (children appraisal, task appraisal) as grouping variables. The Kruskal–Wallis test was used when the grouping variable was language skills (low, moderate, high). Descriptive and inferential analyses were conducted using SPSS software (version 15.0).

## Results

### Analysis of Variations of the Emotional Appraisal Responses

Because it was found that the children appraisal did not coincide with the stimuli valences (task appraisal), an analysis was made considering the accuracy appraisal as a variable of interest. We did not found that this variable was previously explored in the literature. In this way, the valences that children attributed to images were compared (children appraisal compared with task appraisal) and an accuracy appraisal was obtained. Accuracy appraisal varied from 0 to 5 in the negative (*Md* = 2), neutral (*Md* = 2), and positive valences (*Md* = 3).

#### Age Differences

An analysis using the Mann–Whitney *U*-test that compared the accuracy appraisal between ages, showed significant differences between 4- and 5 years olds in the positive (*Z* = −3.14, *p* = 0.002), but not in the negative (*Z* = −1.35, *p* = 0.18) and neutral valences (*Z* = −0.610, *p* = 0.54). The 5 years old children (*M* = 1.74, *SD* = 0.85) were more accurate in the positive valence, compared with the 4 years old children (*M* = 2.27, *SD* = 0.89).

#### Differences by Gender, Language Ability, and Living Conditions at Home

However, the accuracy appraisal did not vary between children with respect to gender (*p* ≥ 0.05), living conditions at home (*p* ≥ 0.05), or language ability (*p* ≥ 0.05) as variables groups.

According to these results, appraisal accuracy should be incorporated into the analyses of expression memory.

### Analysis of Emotional Memory Taking Into Account the Children Appraisal or the Task Appraisal Separately

According to the results described above, children appraisal and task appraisal were compared in the performance of the emotional memory. A global analysis with the Mann–Whitney *U*-Test indicated that the performance memory was significantly different for the negative images (*Z* = −2.03, *p* = 0.04), but it did not differ for neutral images (*Z* = −1.04, *p* = 0.3) and positive images (*Z* = −0.42, *p* = 0.67). In general, children remember more negative images compared to positive and neutral ones.

#### Age Differences

**FIGURE 1:**
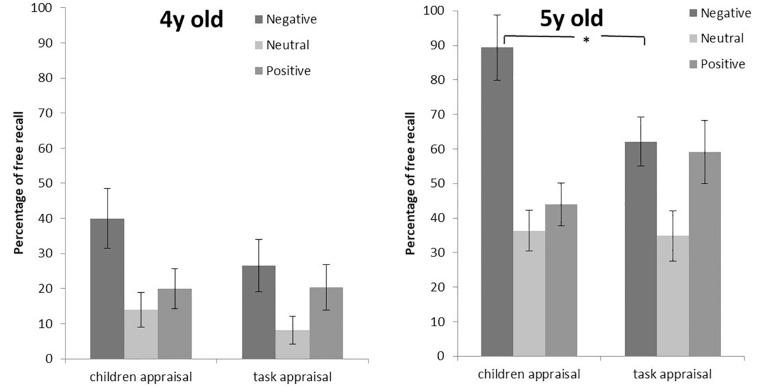
Percentage of negative, neutral, and positive free recall of 4 years old (left) and 5 years old children (right) into account the children appraisal or the task appraisal separately. ^∗^ significant difference relative to negative free recall at *p* = 0.05.

Separated analysis were run for each age group to test differences between children appraisal and task appraisal. Results indicated significant differences only in 5 years olds for negative images (*Z* = −2.00, *p* = 0.04), but not for positive or neutral images (*p* ≥ 0.05). Children who were 5 years old recalled more negative images when they were evaluated taking into account their own assessment than when considering the valence of the task. Figure 1 shows the emotional accuracy of the age groups (4 and 5 years old) to the emotional valence (positive, neutral and negative). Children evoke of negative images was different when comparing children appraisal with task appraisal. Those differences were not evidenced for positive or neutral images.

#### Differences by Gender, Language Skills, and Living Conditions at Home

When we examined the children’s appraisal condition compared with the task appraisal condition as variable group, the Mann–Whitney *U*-Test showed no significant differences in the free recall of negative, neutral, and positive images (*p* ≥ 0.05) for each category of the considered factors (gender, language skills, and living conditions). That is, for boys or girls, from favorable or unfavorable living conditions, and with low, moderate or high language skills, children had similar memories of the three valences regardless of whether we used children appraisal or task appraisal.

## Conclusion and General Discussion

The objective of the present study was to analyze emotional and memory associations during the preschool period. We explored differences in those associations considering individual (i.e., age, gender, language skills) and socio-environmental (i.e., living conditions at homes) factors. We found that appraisal in 4-years-old did not coincide with task appraisal, and that children who were 5-years-old showed greater accuracy appraisal. This differential accuracy is presented in the appraisal of positive visual images. However, the accuracy appraisal did not vary with respect to gender, living conditions at home, or language ability.

With respect to the memory test performance (7–10 days after the session 1), results suggest differences in the number of free recall images between 4 and 5 years old children. In particular, older children evoked more images, regardless their valence. When individual and environmental factors were considered in the performance analysis, no differences were identified. Nevertheless, when the accuracy appraisal (children appraisal or task appraisal) was taken into account, differences in performance were identified for negative stimulus. Five-years-old children evoke more images which they had appraised as negative. This pattern of results provides preliminary evidence regarding the need of including appraisal as a specific aspect of processing in the emotional memory study at these ages. It also suggests that different aspects of emotional memory are possible to be evaluated in young children from different socio-environmental contexts.

Several studies have indicated that the emotional processing of images can generate variations in the consolidation of memory (Channell and Barth, 2013; Kuppens et al., 2013; Leventon and Bauer, 2016). In this sense, those images or events with emotional valences (positive or negative) were remembered more clearly than those with neutral valences (Christianson, 1992; Quas and Lench, 2007; Brainerd et al., 2010; Justel et al., 2013). Our study presents a different pattern of results in relation to other studies (Leventon and Bauer, 2016), in which children evoked images with negative and positive valences in a similar way. One of the differences of the present study is that it analyzes the emotional memory in children with different living conditions, even though the comparisons between the groups of children with favorable and unfavorable conditions were not statistically significant.

Regarding language development and its relation to memory expression, it would be relevant for future studies to analyze vocabulary with and without emotional content in children of these ages, in order to explore whether and how the valences of words are associated with their subsequent.

Higher memory performances of negative visual images when evaluated according to children appraisal may be due to the fact that people usually attribute emotions based on their own emotional states rather than those corresponding to task stimuli (Lerner and Keltner, 2000, 2001). It is also possible that due to this information would be better consolidated and evoked. These differences tend to be less pronounced when children are younger and when memory performance is evaluated according to task appraisal. On the other hand, these differences may be because at this stage the emotional categories are not yet consolidated (Nelson and Russell, 2016), so that children may exhibit variations in their initial appraisal of the stimuli. There are no studies of children that relate these variables, however, Kuppens et al. (2013) studied emotional appraisal of visual stimuli and its relationship with memory processes in adults. They examined how these prominent cognitive and affective components of emotional experience related dynamically to each other in daily life. Their findings revealed insightful patterns of the continuous interplay between core affect and appraisal. That is, appraisals and core affect were not independent properties of emotional experience, but were intricately related in a dynamic interplay that was characterized by congruency between appraisals and valence that suggested a central role in acting and responding to the environment for arousal (Kuppens et al., 2013). Therefore, emotions or moods should not be understood as static phases, but as a dynamic phenomenon that involves components that change and follow each other across time continuously (Kuppens et al., 2009; Boden and Berenbaum, 2010). Based on this reasoning, our study was limited by not including the arousal of visual stimuli. Future studies are proposed to incorporate the evaluation of arousal when analyzing the interaction between emotional appraisal and emotional memory.

On the other hand, appraisal accuracy was surprisingly low for the emotional appraisal task, especially for negative and neutral conditions (performance was around chance level). This brings into question the validity and reliability of this task in assessing emotional appraisal accuracy in 4–5 years olds. This task was used in different studies which reported similar results. However, these findings do not rule out the possibility that these responses are specific to the population that participated in these studies (in all cases, girls and boys residing in Buenos Aires City), and it is possible that cultural factors associated with the expression of these responses could modulate their performance. All of these aspects should be explored in future studies.

The main contribution of our work is the inclusion of the appraisal as an important component to consider in a specific way in the expression of emotional memory of children at this stage of development. The modulation of appraisal on emotional memory opens questions about previous findings in which this component has not been taken in consideration and the implications on the consolidation and retrieval of the memories, especially the negative ones. In this sense, it is important to consider that several studies indicate the prevalence of negative memories during childhood. For example, negative events elicited more false memories than neutral event in a sample of 7-year-old children (Otgaar et al., 2008). In another study, children who developed negative pain memories were more likely to have experienced greater pain intensity and state anxiety during previous painful experiences (Noel et al., 2009, 2012). In a more recent study, emotion influenced the recognition memory of negative scenes in children (Leventon et al., 2014). Other studies provide evidence for negative emotions effects on memory in the school years (Davidson et al., 2006; Cordon et al., 2013). In this sense, the results of the current study also indicate a prevalence of negative memories, however, the performance is greater when evaluating the memories taking into account children‘s appraisal, compared to task appraisal. Thus, our results emphasize the need to include individual differences in emotional appraisal when analyzing the subsequent performance of children.

## Ethics Statement

Informed consents were obtained from parents/caregivers, and ethical approval was obtained from the Ethical Review Committee (Protocol N° 961). This study was conducted in accordance with the American Psychological Association ethical standards, and international and national children rights laws. Once authorization was obtained from the schools where the activities were to be conducted, informative meetings were organized for parents in which they had an opportunity to interact with the research group. The researchers informed them about the objectives and activities of the study, and provided them with an information sheet. Consequently, they were invited to participate and to authorize the participation of their children, which required them to sign an informed consent for each of the evaluations. Likewise, prior to any evaluation, the permission of the children was requested.

## Author Contributions

ER participated as designer, operator, and supervisor, performed the tabulation and prepared the datasets, designed and executed the statistical analysis, and wrote the manuscript. MS and SL participated as designer and coordinator, designed and supervised the statistical analysis, and wrote the manuscript. VR collaborated in preparation of the datasets, statistical analysis design, and manuscript review.

## Conflict of Interest

The authors declare that the research was conducted in the absence of any commercial or financial relationships that could be construed as a potential conflict of interest.

## References

[B1] Aguinaga G. Armentía M. L. Fraile A. Olangua P. Uriz N. (1991). *Prueba de lenguaje Oral de Navarra (PLON). Fondo de Publicaciones del Gobierno de Navarra.* Madrid: TEA.

[B2] Baddeley A. D. (1999). *Essentials of Human Memory.* Abingdon: Routledge.

[B3] Bauer P. J. Doydum A. O. Pathman T. Larkina M. Güler O. E. Burch M. (2012). It’s all about location, location, location: children’s memory for the “where” of personally experienced events. *J. Exp. Child Psychol.* 113 510–522. 10.1016/j.jecp.2012.06.007 23010356 PMC3478447

[B4] Bauer P. J. Larkina M. Deocampo J. (2010). Early memory development. *Wiley Blackwell Handb. Child. Cogn. Dev.* 2 153–179.

[B5] Bermúdez-Rattoni F. Prado-Alcalá R. A. (eds) (2001). *Memoria: Dónde Reside Y Cómo Se Forma.* Mexico: Trillas.

[B6] Boden M. T. Berenbaum H. (2010). The bidirectional relations between affect and belief. *Rev. Gen. Psychol.* 14 227–239. 10.1037/a0019898

[B7] Boltvinik J. (1995). La pobreza en México. I. Metodologías y evolución. *Salud pública de México* 37 288–297. 7502150

[B8] Bradley M. M. Greenwald M. K. Petry M. C. Lang P. J. (1992). Remembering pictures: pleasure and arousal in memory. *J. Exp. Psychol.* 18:379 10.1037//0278-7393.18.2.379 1532823

[B9] Bradley M. M. Lang P. J. (2000). Affective reactions to acoustic stimuli. *Psychophysiology* 37 204–215. 10.1017/s0048577200990012 10731770

[B10] Brainerd C. J. Holliday R. E. Reyna V. F. Yang Y. Toglia M. P. (2010). Developmental reversals in false memory: effects of emotional valence and arousal. *J. Exp. Child Psychol.* 107 137–154. 10.1016/j.jecp.2010.04.013 20547393 PMC2904859

[B11] Brainerd C. J. Stein L. M. Silveira R. A. Rohenkohl G. Reyna V. F. (2008). How does negative emotion cause false memories? *Psychol. Sci.* 19 919–925. 10.1111/j.1467-9280.2008.02177.x 18947358

[B12] Brody L. R. Hall J. A. (2010). “Gender, emotion, and socialization,” in *Handbook of Gender Research in Psychology*, eds Chrisler J. C. McCreary D. R., (New York, NY: Springer), 429–454. 10.1007/978-1-4419-1465-1_21

[B13] Cahill L. Alkire M. T. (2003). Epinephrine enhancement of human memory consolidation: interaction with arousal at encoding. *Neurobiol. Learn. Mem.* 79 194–198. 10.1016/s1074-7427(02)00036-9 12591227

[B14] Canli T. Desmond J. E. Zhao Z. Gabrieli J. D. (2002). Sex differences in the neural basis of emotional memories. *Proc. Natl. Acad. Sci. U.S.A.* 99 10789–10794. 10.1073/pnas.162356599 12145327 PMC125046

[B15] Channell M. M. Barth J. M. (2013). Individual differences in preschoolers’ emotion content memory: the role of emotion knowledge. *J. Exp. Child Psychol.* 115 552–561. 10.1016/j.jecp.2013.01.012 23558117 PMC4782755

[B16] Christianson S. A. (ed.) (1992). “Remembering emotional events: potential mechanisms,” in *The Handbook of Emotion and Memory: Research and Theory*, (Hillsdale, NJ: Lawrence Erlbaum Associates, Inc.), 307–340.

[B17] Cordon I. M. Melinder A. M. Goodman G. S. Edelstein R. S. (2013). Children’s and adults’ memory for emotional pictures: examining age-related patterns using the Developmental Affective Photo System. *J. Exp. Child Psychol.* 114 339–356. 10.1016/j.jecp.2012.08.004 23107226

[B18] Courage M. Cowan N. (eds) (2008). *The Development of Memory in Infancy, and. Childhood.* Hove: Psychology Press.

[B19] Davidson M. C. Amso D. Anderson L. C. Diamond A. (2006). Development of cognitive control and executive functions from 4 to 13 years: evidence from manipulations of memory, inhibition, and task switching. *Neuropsychologia* 44 2037–2078. 10.1016/j.neuropsychologia.2006.02.006 16580701 PMC1513793

[B20] Davies G. M. Logie R. H. (eds) (1993). *Memory in Everyday. Life.* Amsterdam: Elsevier.

[B21] Dennis T. A. Hajcak G. (2009). The late positive potential: a neurophysiological marker for emotion regulation in children. *J. Child Psychol. Psychiatr.* 50 1373–1383. 10.1111/j.1469-7610.2009.02168.x 19754501 PMC3019134

[B22] Ellsworth P. C. Scherer K. R. (2003). Appraisal processes in emotion. *Handb. Affect. Sci.* 572:V595.

[B23] Fernando J. W. Kashima Y. Laham S. M. (2015). Alternatives to the fixed-set model: a review of appraisal models of emotion. *Cogn. Emot.* 31 19–32. 10.1080/02699931.2015.1074548 26291734

[B24] Fidalgo A. M. Tenenbaum H. R. Aznar A. (2018). Are there gender differences in emotion comprehension? Analysis of the test of emotion comprehension. *J. Child Fam. Stud.* 27 1065–1074. 10.1007/s10826-017-0956-5 29576725 PMC5854763

[B25] Fivush R. Baker-Ward L. (2005). The search for meaning: developmental perspectives on internal state language in autobiographical memory. *J. Cogn. Dev.* 6 455–462. 10.1207/s15327647jcd0604_1

[B26] Fivush R. Berlin L. McDermott Sales J. Mennuti-Washburn J. Cassidy J. (2003). Functions of parent-child reminiscing about emotionally negative events. *Memory* 11 179–192. 10.1080/741938209 12820830

[B27] Fivush R. McDermott Sales J. Bohanek J. G. (2008). Meaning making in mothers’ and children’s narratives of emotional events. *Memory* 16 579–594. 10.1080/09658210802150681 18569686

[B28] Ghetti S. Bunge S. A. (2012). Neural changes underlying the development of episodic memory during middle childhood. *Dev. Cogn. Neurosci.* 2 381–395. 10.1016/j.dcn.2012.05.002 22770728 PMC3545705

[B29] Goodman G. S. Quas J. A. Batterman-Faunce J. M. Riddlesberger M. M. Kuhn J. (1994). Predictors of accurate and inaccurate memories of traumatic events experienced in childhood. *Conscious. Cogn.* 3 269–294. 10.1006/ccog.1994.1016

[B30] Goodman G. S. Quas J. A. Batterman-Faunce J. M. Riddlesberger M. M. Kuhn J. (1997). Children’s reactions to and memory for a stressful event: Influences of age, anatomical dolls, knowledge, and parental attachment. *Appl. Dev. Sci.* 1 54–75. 10.1207/s1532480xads0102_1

[B31] Guzmán-Ramos K. Venkataraman A. Morin J. P. Osorio-Gómez D. Bermúdez-Rattoni F. (2018). Differential requirement of de novo Arc protein synthesis in the insular cortex and the amygdala for safe and aversive taste long-term memory formation. *Behav. Brain Res.* 342 89–93. 10.1016/j.bbr.2018.01.006 29326059

[B32] Haden C. A. Ornstein P. A. Eckerman C. O. Didow S. M. (2001). Mother–child conversational interactions as events unfold: linkages to subsequent remembering. *Child Dev.* 72 1016–1031. 10.1111/1467-8624.00332 11480932

[B33] Hudson J. Nelson K. (1986). Repeated encounters of a similar kind: effects of familiarity on children’s autobiographic memory. *Cogn. Dev.* 1 253–271. 10.1016/s0885-2014(86)80004-1

[B34] Justel N. Psyrdellis M. Ruetti E. M. (2013). *Modulación De La Memoria Emocional: Una Revisión De Los Principales Factores Que Afectan Los Recuerdos.* Colombia: Konrad Lorenz University Foundation.

[B35] Kuppens P. Champagne D. Tuerlinckx F. (2012). The dynamic interplay between appraisal and core affect in daily life. *Front. Psychol.* 3:380. 10.3389/fpsyg.2012.00380 23060842 PMC3466066

[B36] Kuppens P. Stouten J. Mesquita B. (2009). Individual differences in emotion components and dynamics: introduction to the special issue. *Cogn. Emot.* 23 1249–1258. 10.1080/02699930902985605

[B37] Kuppens P. Tuerlinckx F. Russell J. A. Barrett L. F. (2013). The relation between valence and arousal in subjective experience. *Psychol. Bull.* 139:917. 10.1037/a0030811 23231533

[B38] Lang P. J. Greenwald M. K. Bradley M. M. Hamm A. O. (1993). Looking at pictures: affective, facial, visceral, and behavioral reactions. *Psychophysiology* 30 261–273. 10.1111/j.1469-8986.1993.tb03352.x 8497555

[B39] Langnes E. Vidal-Pineiro D. Sneve M. H. Amlien I. K. Walhovd K. B. Fjell A. M. (2018). Development and decline of the hippocampal long-axis specialization and differentiation during encoding and retrieval of episodic memories. *Cereb. Cortex* 10.1093/cercor/bhy209 [Epub ahead of print]. 30272128

[B40] Lerner J. S. Keltner D. (2000). Beyond valence: toward a model of emotion-specific influences on judgement and choice. *Cogn. Emot.* 14 473–493. 10.1080/026999300402763

[B41] Lerner J. S. Keltner D. (2001). Fear, anger, and risk. *J. Pers. Soc. Psychol.* 81 146–159. 10.1037//0022-3514.81.1.146 11474720

[B42] Leventon J. S. Bauer P. J. (2016). Emotion regulation during the encoding of emotional stimuli: effects on subsequent memory. *J. Exp. Child Psychol.* 142 312–333. 10.1016/j.jecp.2015.09.024 26597138

[B43] Leventon J. S. Stevens J. S. Bauer P. J. (2014). Development in the neurophysiology of emotion processing and memory in school-age children. *Dev. Cogn. Neurosci.* 10 21–33. 10.1016/j.dcn.2014.07.007 25160677 PMC6987950

[B44] Lipina S. J. Martelli M. I. Vuelta B. Injoque-Ricle I. Colombo J. A. (2004). Pobreza y desempeño ejecutivo en alumnos preescolares de la ciudad de Buenos Aires (Argentina). *Interdisciplinaria* 21 53–93.

[B45] Lipina S. J. Martelli M. I. Vuelta B. L. Colombo J. A. (2005). Performance on the AnoB task of argentinean infants from unsatisfied basic needs homes. *Interam. J. Psychol.* 39 49–60.

[B46] McManis M. H. Bradley M. M. Berg W. K. Cuthbert B. N. Lang P. J. (2011). Emotional reactions in children: verbal, physiological and behavioral responses to affective pictures. *Psychophysiology* 38 222–231. 10.1017/s0048577201991140 11347868

[B47] Merritt K. A. Ornstein P. A. Spicker B. (1994). Children’s memory for a salient medical procedure: implications for testimony. *Pediatrics* 94 17–23. 8008531

[B48] Montirosso R. Peverelli M. Frigerio E. Crespi M. Borgatti R. (2010). The development of dynamic facial expression recognition at different intensities in 4- to 18-year-olds. *Soc. Dev.* 19 71–92. 10.1111/j.1467-9507.2008.00527.x

[B49] Moors A. (2013). On the causal role of appraisal in emotion. *Emot. Rev.* 5 132–140. 10.1177/1754073912463601

[B50] Moors A. (2017). The integrated theory of emotional behavior follows a radically goal-directed approach. *Psychol. Inq.* 28 68–75. 10.1080/1047840x.2017.1275207

[B51] Moors A. Scherer K. R. (2013). “The role of appraisal in emotion,” in *Handbook of Cognition and Emotion*, eds Robinson M. Watkins E. Harmon-Jones E. (New York, NY: Guilford Press).

[B52] Nelson N. L. Russell J. A. (2016). Building emotion categories: children use a process of elimination when they encounter novel expressions. *J. Exp. Child Psychol.* 151 120–130. 10.1016/j.jecp.2016.02.012 27222441

[B53] Nieto M. A. P. Delgado M. M. R. (2006). Procesos de valoración y emoción: caracterís-ticas, desarrollo, clasificación y estado actual. *REME* 9:8.

[B54] Noel M. Chambers C. T. McGrath P. J. Klein R. M. Stewart S. H. (2012). The influence of children’s pain memories on subsequent pain experience. *Pain* 153 1563–1572. 10.1016/j.pain.2012.02.020 22560288

[B55] Noel M. McMurtry C. M. Chambers C. T. McGrath P. J. (2009). Children’s memory for painful procedures: the relationship of pain intensity, anxiety, and adult behaviors to subsequent recall. *J. Pediatr. Psychol.* 35 626–636. 10.1093/jpepsy/jsp096 19889718

[B56] Ohrmann P. Pedersen A. Braun M. Bauer J. Kugel H. Kersting A. (2010). Effect of gender on processing threat-related stimuli in patients with panic disorder: sex does matter. *Depress. Anxiety* 27 1034–1043. 10.1002/da.20721 20602432

[B57] Otgaar H. Candel I. Merckelbach H. (2008). Children’s false memories: easier to elicit for a negative than for a neutral event. *Acta Psychol.* 128 350–354. 10.1016/j.actpsy.2008.03.009 18462700

[B58] Parsafar P. Davis E. L. (2018). Intrapersonal emotion regulation processes influence what children remember about their emotional experiences. *Child Dev.* 10.1111/cdev.13070 [Epub ahead of print]. 29660774

[B59] Prats L. Segretin M. S. Fracchia C. Kamienkowski J. Pietto M. Hermida J. (2017). Asociaciones entre factores individuales y contextuales con el desempeño cognitivo en preescolares de hogares con Necesidades Básicas Insatisfechas (NBI). *Cuadernos de Neuropsicología/Panamerican J. Neuro.* 11 42–77. 10.7714/CNPS/11.2.201

[B60] Principe G. F. Trumbull J. Gardner G. Van Horn E. Dean A. M. (2017). The role of maternal elaborative structure and control in children’s memory and suggestibility for a past event. *J. Exp. Child Psychol.* 163 15–31. 10.1016/j.jecp.2017.06.001 28734134

[B61] Quas J. A. Lench H. C. (2007). Arousal at encoding, arousal at retrieval, interviewer support, and children’s memory for a mild stressor. *Appl. Cogn. Psychol.* 21 289–305. 10.1002/acp.1279

[B62] Rodríguez S. M. Schafé G. E. LeDoux J. E. (2004). Molecular mechanisms underlying emotional learning and memory in the lateral amygdala. *Neuron* 44 75–91. 10.1016/j.neuron.2004.09.014 15450161

[B63] Roseman I. J. Smith C. A. (2001). “Appraisal theory: overview, assumptions, varieties, controversies,” in *Series in Affective Science. Appraisal Processes in Emotion: Theory, Methods, Research*, eds Scherer K. R. Schorr A. Johnstone T., (New York, NY: Oxford University Press), 3–19.

[B64] Russell J. A. (2009). Emotion, core affect, and psychological construction. *Cogn. Emot.* 23 1259–1283. 10.1080/02699930902809375

[B65] Salmon K. Price M. Pereira J. K. (2002). Factors associated with young children’s long-term recall of an invasive medical procedure: a preliminary investigation. *J. Dev. Behav. Pediatr.* 23 347–352. 10.1097/00004703-200210000-00008 12394523

[B66] Scherer K. R. Fontaine J. R. (2018). The semantic structure of emotion words across languages is consistent with componential appraisal models of emotion. *Cogn. Emot.* 33 673–682. 10.1080/02699931.2018.1481369 29855214

[B67] Segretin M. S. Lipina S. J. Hermida M. J. Sheffield T. Nelson J. M. Espy K. A. (2014). Predictors of cognitive enhancement after training in a sample of Argentinean preschoolers from diverse socioeconomic backgrounds. *Front. Dev. Psychol.* 13:A205. 10.3389/fpsyg.2014.00205 PMC3952047 24659975

[B68] Segretin M. S. Lipina S. J. Petetta D. R. (2009). Consideraciones metodológicas y conceptuales para el análisis de predicción del desempeño escolar en base a indicadores del contexto hogareño y escolar”. *Rev. Iberoam. Eva luación Educ.* 2 104–123.

[B69] Siller-Pérez C. Fuentes-Ibañez A. Sotelo-Barrera E. L. Serafín N. Prado-Alcalá R. A. Campolongo P. (2018). Glucocorticoid interactions with the dorsal striatal endocannabinoid system in regulating inhibitory avoidance memory. *Psychoneuroendocrinology* 99 97–103. 10.1016/j.psyneuen.2018.08.021 30216767

[B70] Tessler M. Nelson K. (1994). Making memories: the influence of joint encoding on later recall by young children. *Conscious. Cogn.* 3 307–326. 10.1006/ccog.1994.1018

[B71] Wang Q. (2008). Emotion knowledge and autobiographical memory across the preschool years: a cross-cultural longitudinal investigation. *Cognition* 108 117–135. 10.1016/j.cognition.2008.02.002 18353299

[B72] Widen S. C. Russell J. A. (2010). The “Disgust Face” conveys anger to children. *Emotion* 10 455–466. 10.1016/j.msard.2019.05.029 20677863

